# Research Progress on the Mechanism of Action of Food-Derived ACE-Inhibitory Peptides

**DOI:** 10.3390/life15081219

**Published:** 2025-08-01

**Authors:** Ting Li, Wanjia Du, Huiyan Huang, Luzhang Wan, Chenglong Shang, Xue Mao, Xianghui Kong

**Affiliations:** 1 Institute of Microbiology Heilongjiang Academy of Sciences, Harbin 150010, China; hyskoat@163.com (T.L.); duwj010708@163.com (W.D.); hhy244025132@163.com (H.H.); 2 Shandong Academy of Agricultural Sciences, Jinan 250100, China; wanluzhang657@163.com; 3 Shandong Dongping Kehai Mushroom Industry Co., Taian 271500, China; zhongxin0538@163.com; 4 College of Agriculture and Biology, Liaocheng University, Liaocheng 252059, China

**Keywords:** angiotensin converting enzyme, angiotensin converting enzyme-inhibitory peptide, mechanism of action

## Abstract

Hypertension is a major pathogenic contributor to cardiovascular diseases, primarily mediated through activation of the angiotensin-converting enzyme (ACE) system. Food-derived ACE-inhibitory peptides represent a promising alternative to synthetic drugs due to their favorable safety profile and minimal side effects. ACE-inhibitory peptides have been extensively identified from various foods, with their antihypertensive activity and molecular mechanisms comprehensively characterized through in vitro and in vivo studies. ACE-inhibitory peptides can be prepared by methods such as natural extraction, enzymatic hydrolysis, and fermentation. The production process significantly modulates structural characteristics of the polypeptides including peptide chain length, amino acid composition, and sequence, consequently determining their functional activity. To comprehensively elucidate the gastrointestinal stability and mechanisms action of ACE-inhibitory peptides, integrated experimental approaches combining both in vitro and in vivo methodologies are essential. This review systematically examines current advances in food-derived ACE-inhibitory peptides in terms of sources, production, structure, in vivo and in vitro activities, and bioavailability.

## 1. Introduction

According to data from the World Health Organization (WHO), approximately 1.28 billion adults aged 30 to 79 years worldwide suffered from hypertension in 2023. Hypertension and its complications are leading causes of death in the global population [1]. Hypertension is a complex, multifactorial chronic pathophysiological disease characterized not only by elevated blood pressure but also by functional and structural abnormalities in the heart and vasculature. It is characterized by elevated systemic arterial pressure, which causes progressive dysfunction and damages to target organs (including the heart, kidneys, brain, and vasculature), ultimately leading to severe cardiovascular diseases complications such as stroke, heart failure, and renal failures [2,3]. Angiotensin-converting enzyme (ACE) catalyzes the conversion of inactive angiotensin I (Ang I, Asp-Arg-Val-Tyr-Ile-His-Leu-Val-Ile-His, a decapeptide) into the highly potent vasoconstrictor angiotensin II (Ang II, Asp-Arg-Val-Tyr-Ile-His-Pro-Phe, a octapeptide). This process also stimulates aldosterone release, collectively contributing to increased blood pressure. Meanwhile, ACE (acting as kininase II) degrades bradykinin, thereby inhibiting its vasodilative effect and further contributing to elevated blood pressure [4,5]. Therefore, effectively inhibiting ACE activity has emerged as a key therapeutic strategy for managing and treating hypertension [6]. Antihypertensive drugs, especially ACE inhibitors, are essential in hypertension management [7,8].

Antihypertensive drugs may be administered as monotherapy or combination therapy, depending on disease severity and drug class characteristics [9]. Clinically used synthetic ACE inhibitors, such as captopril (Table 1), demonstrate significant antihypertensive efficacy. However, long-term administration is associated with adverse effects ranging from mild (cough, fatigue) to severe (angioedema, hyperkalemia), along with potential drug interaction when combined with other medications [6]. Consequently, food-derived antihypertensive peptides have emerged as a promising research focus, offering advantages including high safety, absence of adverse effects in normotensive individuals, and suitability for chronic administration [10]. Table 2 provides a summary of studies on anti-ACE peptides from different sources. ACE-inhibitory polypeptides from various sources demonstrate distinct inhibitory modalities, including competitive, non-competitive, uncompetitive, and mixed inhibition kinetics against the enzyme [11]. Elucidating the inhibition modality of polypeptides against ACE provides critical insights into their interference with the key steps of ACE catalytic mechanisms [12,13,14,15]. Food-derived peptides primarily exhibit competitive inhibition against ACE, as demonstrated by kinetic analyses [13,16]. The structural characteristics and ACE-inhibitory efficacy of food-derived polypeptides are critically determined by extraction, separation, and purification processes, which collectively modulate the structural integrity and functional performance of the final products [4,17].

This review synthesizes contemporary research on food-derived ACE-inhibitory peptides, addressing (1) innovative extraction and purification strategies, (2) structural determinants of bioactivity, and (3) in vitro/in vivo evidence of antihypertensive effects and molecular mechanisms.

## 2. Materials and Methods

We systematically searched PubMed and CNKI (2000–2025) using the terms “Angiotensin-I-converting enzyme” and “ACE Inhibitory Peptide” to identify relevant in vitro, in vivo, and clinical studies on ACE inhibition. After duplicate removal, we screened titles/abstracts and selected articles for full-text review. Final inclusion was based on studies’ contributions to understanding ACE inhibition mechanisms, peptide sources (food-derived/synthetic), and therapeutic potential. Key mechanisms were visualized using BioRender (Available online: https://www.biorender.com/ (accessed on 16 June 2025)), including ACE inhibition pathways, peptide preparation processes, and 3D molecular docking structures.

## 3. Angiotensin-Converting Enzyme (ACE) and Its Inhibitory Mechanisms

**Figure 1:**
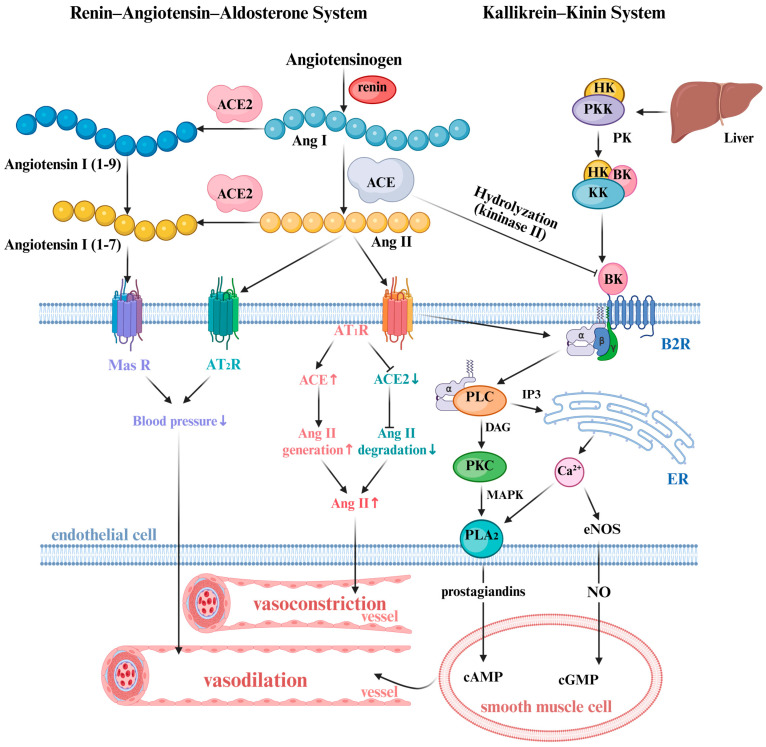
Schematic representation of the Renin–Angiotensin–Aldosterone System and Kallikrein–Kinin System with physiological interrelation [7,48,55]. PKK: prekallikrein; HK: high-molecular-weight kininogen; PK: plasma kallikrein, BK: bradykinin; PLC: phospholipase C; IP3: inositol 1,4,5-triphosphate; ER: endothelial reticulum; DAG: diacylglycerol; MAPK: mitogen-activated protein kinase, PLA2: phospholipase A2; NO: nitric oxyde; eNOS: endothelial NO synthase.

ACE is a zinc-dependent carboxydipeptidase containing two homologous domains (N- and C-domain), each featuring a catalytically active zinc-binding site. It composed of galactose, fructose, mannose, N-acetylneuraminic acid, and N-acetylglucosamine [17,36,37]. The molecular weight of ACE typically ranges from 130 to 170 kDa, with variation determined by isoform type and the degree of glycosylation of ACE [36]. ACE regulates blood pressure through dual participation in both the Renin–Angiotensin–Aldosterone System (RAAS) and Kallikrein–Kinin System (KKS), as illustrated in Figure 1.

Following its secretion by the kidneys, renin catalyzes the conversion of angiotensinogen into inactive Ang-I, which is subsequently cleaved by pulmonary ACE into the vasoconstrictor Ang-II; as the primary effector molecule of RAAS, Ang-II induces vascular dysfunction by stimulating oxidative stress through superoxide anions (O_2_^−^), peroxynitrite (OONO^−^), and hydrogen peroxide (H_2_O_2_), triggering potent vascular constriction, promoting inflammation via macrophage and monocyte activation, and mediating inflammation [38,39,40]. The KKS consists of kallikrein, kininogen, kinins, kininase, and kinin receptors, functioning primarily to counterbalance the detrimental effects of RAAS on target organs and functions [41]. During vascular inflammation, activation of the KKS triggers proteolytic cleavage of high-molecular-weight kininogen, releasing kinins such as bradykinin (BK). BK binds to the B2R kinin receptor on vascular endothelial cell membranes, initiating G protein-coupled receptor (GPCR) signaling through phospholipase C (PLC) activation. This signaling cascade stimulates endothelial cells to release vasoactive mediators like nitric oxide (NO) and prostaglandins [42,43,44,45,46]. Both NO and some prostaglandins function as potent counterregulators of Ang-II-induced vasoconstriction [42,46,47]. Meanwhile, ACE also functions as kininase II, which exhibits high catalytic efficiency for BK degradation, cleaving two distinct bonds at the C-terminus, indirectly enhancing the vasoconstrictive effect of Ang-II [48]. In addition to suppressing Ang-II synthesis via ACE inhibition, ACE inhibitors exert direct pharmacological effects through B2R activation. They increase peptides such as kinins, N-acetyl-seryl-aspartyl-lysyl-proline (Ac-SDKP), and ANG 1–7, which may contribute to their antihypertensive effects and cardiovascular and renal protective effects, promoting vasodilation and reducing peripheral vascular resistance [49,50].

The sequence structure of ACE-inhibitory peptides can be identified through in vitro enzymatic hydrolysis of precursor proteins or in vivo gastrointestinal digestion processes [51]. Furthermore, molecular docking simulations can predict the binding conformations of food-derived ACE-inhibitory peptides at the active sites of ACE, thereby elucidating the molecular mechanisms through which food-derived ACE-inhibitory peptides interact with their target protein ACE and their potential therapeutic effects [52]. ACE-inhibitory peptides are inherently encrypted within the primary structure of precursor proteins and remain inactive in this stage; these inactive polypeptide sequences can be released through either thermal processing or enzymatic hydrolysis [53]. The structural integrity and bioactivity of ACE-inhibitory peptides are significantly influenced by both their protein sources and the purification methodologies employed during preparation [54].

## 4. Production of Food-Derived ACE-Inhibitory Peptides

**Figure 2:**
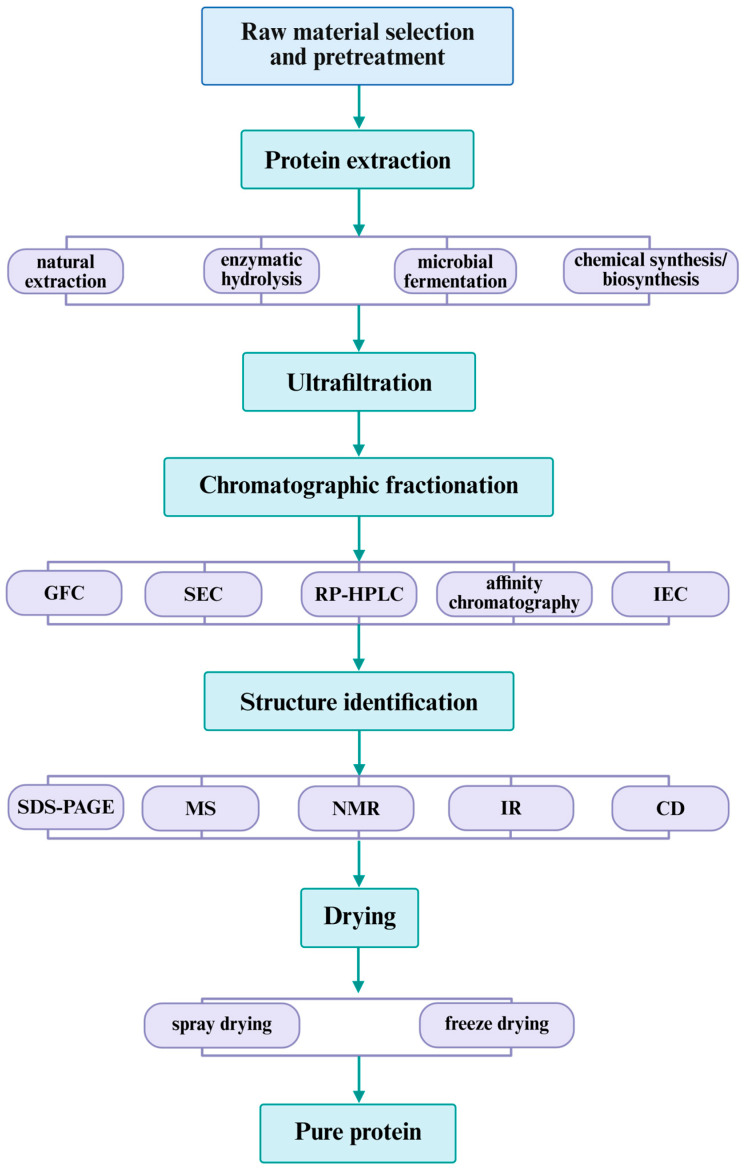
The preparation process of ACE-inhibitory peptides. GFC: gel filtration chromatography; SEC: size exclusion chromatography; RP-HPLC: reverse-phase high-performance chromatography; IEC: ion exchange chromatography; SDS-PAGE: sodium dodecyl sulfate polyacrylamide gel electrophoresis; MS: mass spectrometry; NMR: nuclear magnetic resonance; IR: infrared spectroscopy; CD: circular dichroism.

The methods for isolating ACE-inhibitory peptides exhibit significant variation across different protein sources, primarily due to inherent differences in protein composition and structural differences; the primary methodologies include natural extraction, enzymatic hydrolysis, microbial fermentation, and chemical synthesis or recombinant biosynthesis [4,56,57,58]. The preparation workflows for food-derived ACE-inhibitory peptides from various protein sources are summarized in Figure 2. Natural extraction denotes the method of directly separating and enriching bioactive peptides from native protein matrices through physical, chemical, or biological means, including solvent extraction, adsorption separation, and other methods [57,59]. Meng et al. isolated ACE-inhibitory peptides from walnut protein through an alkali-soluble acid-precipitation approach coupled with enzymatic hydrolysis [60]. With the degree of hydrolysis (DH) and ACE inhibition rate as evaluation indices, the preparation process and parameters were optimized via single-factor tests and response surface methodology (RSM). The optimal hydrolysis conditions were determined as a pH of 9.10, 54.50 °C, and a reaction time of 136 min. Under these conditions, the hydrolysate exhibited a maximum ACE inhibition rate of 63.93% [60]. Solvent extraction has been successfully employed for the isolation of plant-derived ACE-inhibitory peptides. Common extraction solvents include methanol, ethanol, and water, and the extraction yield of polypeptides varies depending on botanical source, solvent composition, and mixing ratios [4,5,23]. Enzymatic hydrolysis represents a widely employed method for food-derived antihypertensive hydrolysate containing ACE-inhibitory peptides. Diverse proteases sources, including fermentation-derived enzymes, have been extensively utilized for this purpose (Table 2). Microbial fermentation has emerged as the preferred method for generating bioactive peptides from dairy and fermented food products, which is beneficial for industrial-scale production by using the proteolytic activity of probiotic starter cultures [61]. Chemical and biosynthetic approaches to polypeptides production facilitate systematic screening of ACE-inhibitory peptides while enabling comprehensive investigation of their sequence characteristics and molecular mechanisms [58]. Ma et al. conducted a comprehensive computational analysis, employing molecular docking to systematically evaluate interaction mechanisms between 160,000 tetrapeptides. The study identified t Tyr, Phe, His, and Arg as characteristic amino acids in ACE-inhibitory peptides, particularly Trp [58].

### 4.1. Enzymatic Hydrolysis for Obtaining ACE-Inhibitory Peptides from Various Foods

Enzymatic hydrolysis has become the predominant method for obtaining antihypertensive peptides from food proteins, owing to its specificity and efficient release of target bioactive peptides. Multiple enzymes are utilized in the production of antihypertensive peptides, including alkaline protease, flavor protease, trypsin, pepsin, papain, neutral protease, and compound protease [10,22,30,33,62]. Depending on the specific enzyme-substrate combination, enzymatic reactions yield functionally distinct end products. The substrate specificity of proteases can significantly determine the peptide profile and biological functionality of protein hydrolysates. The amino acid composition of precursor proteins and the structural properties of peptide bonds determine the optimal enzyme selection for generating bioactive peptides with desired functions. Optimal enzymatic hydrolysis requires careful selection of enzymes according to the specific protein substrate characteristics [17,61]. In a review by Ceren Daskaya-Dikmen et al., they systematically compared the ACE-inhibitory activities of peptides derived from plant protein hydrolysis using various proteolytic enzymes. The study showed that Alcalase and Thermolysin were particularly effective in generating ACE-inhibitory peptides with higher activity, with significantly lower IC_50_ values indicating greater potency [4]. Nancy Goyal et al. hydrolyzed moth bean protein using Alcalase, papain, and trypsin. Based on ACE-inhibitory activity screening, Alcalase hydrolysates demonstrated superior bioactivity and further separated them by ultrafiltration [63]. Bao et al. conducted a comparative study utilizing four distinct proteolytic enzymes—alkaline protease, flavor protease, neutral protease, and proteinase K—to screen for more active ACE-inhibitory peptides from defatted goat and cow milk. The results showed that alkaline protease treatment produced goat milk hydrolysates with remarkable ACE-inhibitory activity of 95.31%, whereas proteinase K showed optimal performance in hydrolyzing cow milk with 81.28% ACE inhibition [64]. The review by Du-Min Jo et al. highlighted that different fish proteins, such as those of skipjack tuna, Alaska pollock, and monkfish, were subjected to enzymatic hydrolysis using alkaline protease, pepsin, and trypsin to obtain highly effective ACE-inhibitory peptides [52].

Beyond enzyme specificity, key enzymatic hydrolysis parameters such as temperature, duration, pH, and the enzyme-to-substrate ratio critically govern the structure and yield of ACE-inhibitory peptides, thereby modulating their activity. Notably, multi-enzyme systems may demonstrate more enhanced ACE-inhibitory activity than single-enzyme treatments [4]. Lu et al. systematically evaluated the performance of various proteases in hydrolyzing sesame protein to produce ACE-inhibitory peptides and identifying a binary enzyme system (alkaline protease: trypsin = 3:7 activity unit ratio) through comprehensive screening. Process optimization revealed that the optimal conditions (pH of 8.35, enzyme-to-substrate ratio of 6145 U/g, hydrolysis duration of 4.4 h) yielded a peak ACE-inhibitory activity of 98.10% [65]. Kumar et al. employed a Box–Behnken experimental design coupled with RSM to systematically optimize key hydrolysis parameters including alkaline protease dosage, incubation temperature, duration, and substrate-to-liquid (S/L) ratio for maximal peptide recovery from rohu fish processing byproducts. The RSM-optimized conditions for maximal ACE-inhibitory peptide production comprised a 1.08% (*v*/*w*) alkaline protease dosage, 52.10 °C incubation temperature, 129.18 min hydrolysis duration, and 0.8:1 S/L ratio [66]. Zheng et al. identified alkaline protease as the most effective enzyme among five proteases for hydrolyze skipjack tuna muscle, followed by a hybrid optimization approach combining single-factor and response surface methodology. Under the optimized conditions (pH of 9.4, 2.3% enzyme loading, 56.2 °C), the hydrolysate exhibited peak ACE-inhibitory activity (72.71%) at 1.0 mg/mL [67].

### 4.2. Fermentation Process

ACE-inhibitory peptides may alternatively be produced via microbial fermentation using protease-producing strains. Microbial fermentation process harnesses endogenous proteolytic enzyme production by microorganisms, enabling targeted proteins hydrolysis into bioactive peptides [68]. A broad protease spectrum is produced by microbial consortia, and fermentation can yield structurally diverse polypeptides with different chain lengths and sequences [69]. To maximize bioactive peptides and enhance their biological activity, rational starter culture selection is essential [70]. Lactic acid bacteria (LAB) represent prevalent starter cultures due to their biosynthetic capacity to produce important metabolites [71]. Strains such as *Lactobacillus rhamnosus* and *Lactobacillus delbrueckii* in the genus *Lactobacillus*, *Saccharomyces cerevisiae* in the genus *Saccharomyces*, and *Bacillus natto* in the genus *Bacillus* can all be used as starters to produce ACE-inhibitory peptides [68,72]. Fermentation parameters, such as inoculum density, crucially influence the biosynthesis of ACE-inhibitory peptides. Puspitojati et al. demonstrated a positive correlation between the activity of ACE-inhibitory peptides and fermentation duration in fungal fermentation systems. Meanwhile, the low-molecular-weight (LMW) peptide fraction demonstrated superior ACE-inhibitory activity [73]. Xu et al. developed an optimized fermentation protocol using selenium-enriched *Bacillus natto* to bio-transform chickpea proteins into ACE-inhibitory peptides. Under nutrient-replete conditions, a positive correlation was observed between ACE-inhibitory activity and inoculation density [72]. In addition, co-culture of strains can also affect ACE-inhibitory activity. Shahram et al. investigated both single and co-culture fermentation systems using *Lactobacillus delberueckiisubsp.bulgaricus*, *Limosilactobacillusreuteri*, and *Lactococcus lactissubsp. Lactis* as starters for milk fermentation. Following seven days of fermentation with subsequent refrigeration, the triple-fermented milk exhibited peak proteolytic rate and ACE-inhibitory activity (IC_50_ = 0.61 mg/mL) [74]. Heydarian et al. established a *Lactobacillus fermentum*-*Saccharomyces cerevisiae* co-culture system using whey sludge substrate. Through the factorial design of culture media and optimization of critical parameters (temperature, pH, inoculation ratio, and quantity), they generated bioactive peptide-enriched extracts. The bioactive peptides in the fermented extracts were fractionated, identified, and quantified using analytical platforms including HPLC and MS. The results showed that optimal C/N ratio significantly promoted the production of bioactive peptides [68].

## 5. Isolation, Purification, and Sequencing of Peptides

Food-derived ACE-inhibitory peptides often exist as crude peptides with suboptimal purity and require downstream processing including separation and purification. UF employs semi-permeable membranes with different pore sizes and molecular weight cut-offs (MWCOs) for preliminary fractionation of suspended particulates. This technique enables molecular weight-based fractionation to collect peptide fractions within the target range [73,75].

UF serves as a pre-fractionation step before chromatographic fractionation, enabling samples’ concentration and purification; it is predominantly combined with other fractionation techniques [13,25,36,76]. Plant-derived protein hydrolysates demonstrate a more constrained molecular weight (MW) distribution (typically 1–10 kDa), while animal counterparts exhibit greater dispersity (1.0–30 kDa). However, smaller peptides typically possess higher biological activity [17,59,72,77]. Hydrolysate antihypertensive activity exhibits time-dependent enhancement, attributable to progressive accumulation of low-MW peptides during extended processing [65]. Chromatographic techniques used for further purification typically include size exclusion chromatography (SEC), reverse-phase high-performance liquid chromatography (RP-HPLC), affinity chromatography, gel filtration chromatography (GFC), etc. [12,30,78]. Purified proteins exhibit marked heterogeneity in MW, chain length, structure, and amino acid composition. These variations confer unique physicochemical properties upon polypeptides, thereby modulating their biological activities. Based on the MW of ACE-inhibitory peptides, their binding affinity for ACE can be determined. High-MW peptides may exhibit steric hindrance at ACE’s catalytic pockets, but ACE-inhibitory activity does not entirely depend on the MW of polypeptides [79]. Nasir et al. performed sequential ultrafiltration of shortfin scad muscle protein hydrolysate, isolating distinct MW fractions (<3 kDa, <5 kDa, and <10 kDa). The <3 kDa fraction exhibited optimal ACE-inhibitory activity (77.04%), with a significant inverse correlation observed between MW and bioactivity [80].

After purification, drying techniques are essential to enhance product shelf-life while preserving bioactive constituents and ACE-inhibitory activity, followed by quantitative assessment. Spray drying (SD) and freeze drying (FD) are the predominant common methods for producing stable enzymatic hydrolysate powders [81,82]. Ye et al. investigated a comprehensive characterization of protein hydrolysate from bighead carp skins dried by FD and SD. The results showed that the drying techniques affected the physicochemical characteristics of protein products, but they exhibited no statistically significant influence on ACE-inhibitory activity [81]. Purified peptides’ structures need to be identified and characterized through SDS-PAGE, MS, and protein sequencing to determine molecular mass, amino acid residue composition, and sequences [12,13,21,22,62]. Studies have shown that purification protocols can potentiate the anti-ACE activity of products. Hu et al. used four distinct proteases to hydrolyze *Lepidotriglamicroptera* and employed multistage purification methods such as UF, GFC, and prep-HPLC. The results showed that after separation by a Sephadex G-15 column, the lowest IC_50_ value reached 0.38 mg/mL. Subsequently, prep-HPLC reduced the IC_50_ value of the product to 0.28 mg/mL, indicating an improvement in bioactivity [83]. Guangqiang Wei et al. valorized Rushan cheese by-products to generate ACE-inhibitory peptides. Following enzymatic hydrolysis, ultrafiltration (UF), and liquid chromatography tandem mass spectrometry (LC-MS/MS), two novel ACE-inhibitory peptides (FDRPFL and KWEKPF) were characterized. Kinetic characterization via Lineweaver–Burk plots revealed that they exhibited non-competitive and mixed inhibition patterns. Molecular docking and molecular dynamics (MD) simulations indicated that both peptides form stable complexes with ACE through multiple intermolecular interactions [84].

## 6. Structure–Activity Relationship

**Figure 3:**
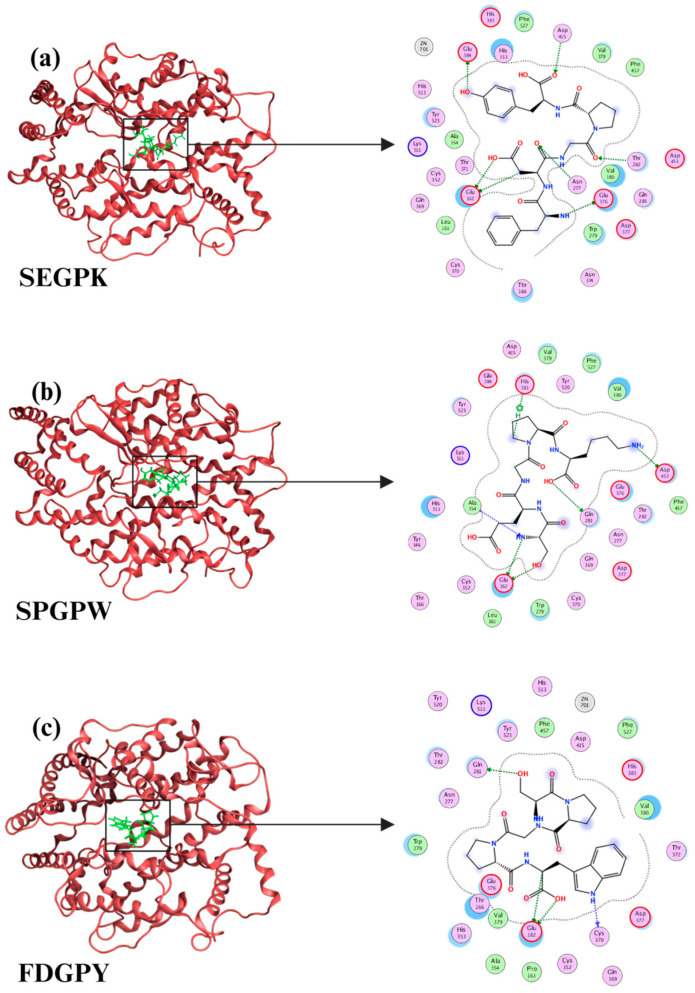
Three-dimensional structural diagram of the molecular docking results between human ACE and ACE-inhibitory peptides. Ser-Glu-Gly-Pro-Lys occupies the active region around amino acids such as His 383, Glu 384, and Asp 415. Ser-Pro-Gly-Leu-Trp occupies the active region around amino acids such as His 383, Glu 376, and Asp 453. Phe-Asp-Gly-Pro-Tyr occupies the active region around amino acids such as His 383, Glu 162, and Asp 377. Adapted and modified from Hu, Y.-D. et al. [10].

The molecular structure of ACE-inhibitory peptides significantly influences their interaction with the active site of ACE, consequently modulating inhibitory activity against ACE [79]. Hu et al. used in silico simulations to elucidate that the activity of marine-derived ACE-inhibitory peptides mediate their effects through hydrogen bonds, hydrophobic interactions, and electrostatic forces, as shown in Figure 3 [10]. Processing methodologies can induce structural polymorphisms that govern peptide activity, including peptide chain length, peptide hydrophobicity, amino acid (aa) residue types, and sequences, all modulating biological activity [79,85]. Peptide chains length appears to be directly correlated with ACE-inhibitory activity. Potent ACE-inhibitory peptides are typically short-chain peptides consisting of 2 to 12 amino acid residues [86]. Given the narrow binding channel of ACE for high-MW peptides, elongated peptide chains reduce active-site accessibility [87].

However, residue-specific physicochemical properties may be more relevant than chain length [88]. ACE features two homologous catalytic domains (N- and C-domains). Following docking into the ACE channel, they establish stable complexes through low-barrier hydrogen bonds and hydrophobic interactions, indirectly inducing allosteric conformational shifts to hinder substrate (Ang-I) access to entering the catalytic center [87,89]. Generally, polypeptides containing aliphatic/aromatic hydrophobic amino acids, branched cationic side chains, and proline at the C-terminus exhibit dual renin and ACE inhibition with lower IC_50_ values [79]. Studies have shown that bulky amino acids with larger side chains (Tyr, Phe, His, Arg, and especially Trp) and hydrophobic amino acids (Ala, Val, Leu, Pro, and Glu) constitute critical pharmacophores for ACE inhibition [58,86,88]. Hydrophobic amino acids mediate high-affinity anchoring within ACE. The β-carboxylate groups of Asp and Glu coordinate to chelate Zn^2+^ ions, thereby abolishing ACE’s enzymatic activity through active-site blockade. Additionally, the dicarboxylate moieties Asp and Glu readily form hydrogen bonds [52,88,90,91]. Furthermore, Phe, Trp, Tyr, and Val synergize with proline at C-terminus cationic character to promote the hydrolysis of Ang-I, thereby enhancing the ACE-inhibitory activity of peptides [90].

Biochemical evidence demonstrates that isofunctional catalytic competence between the two domains of ACE exhibit the same affinity for Ang-I and equivalent proteolytic efficiency toward BK [92,93]. Upon selective inhibition of the N-domain catalytic center of ACE in somatic cells, the conversion of Ang-I to Ang-II occurs without significant attenuation. In contrast, targeted inhibition C-domain abolishes Ang-II production [92,94]. This indicates that while both catalytic centers of ACE in somatic cells are involved in Ang-I hydrolysis, the conversion of Ang-I to Ang-II relies exclusively on its C-domain. Activation of the C-domain is closely correlated with elevated blood pressure. Thus, ACE C-domain-specific inhibitors may represent an effective in hypertension management [92]. Studies indicate that active peptides containing C-terminal Phe, Pro, and Tyr residues exhibit relatively high binding affinity for the ACE active site [17,90]. Song et al. identified food-derived dipeptides AY (Ala-Tyr), LY (Leu-Tyr), and IY (Ile-Tyr) as selective C-domain ACE inhibitors using domain-specific fluorescent substrates, consistently with these findings [93]. The review by Olalere et al. further demonstrates that ACE preferentially recognizes hydrophobic amino acid residues at the C-terminal of active peptides, with C-terminal proline residues representing a key structural feature of potent ACE-inhibitory dipeptides and tripeptides [51]. Extensive research has demonstrated that most food-derived ACE-inhibitory peptides feature a C-terminal proline residue [95].

## 7. Gastrointestinal Digestive Stability and Bioavailability of ACE-Inhibitory Peptides

### 7.1. In Vitro Studies

The gastrointestinal stability of ACE-inhibitory peptides is essential for maintaining their biological efficacy. For ACE-inhibitory peptides to exert their effects after intestinal absorption, key amino acids must resist enzymatic hydrolysis during digestion [96]. Research has demonstrated that the gastrointestinal stability of ACE-inhibitory peptides varies significantly across different peptide sequences. Consequently, it is essential to identify enzymatically stable peptide segments that retain high ACE-inhibitory activity through experimental methods [4]. Static in vitro digestion models typically include two or three phases to simulate the digestive processes occurring in the oral cavity, stomach, and small intestine. Proteolytic enzymes including pepsin, trypsin, and chymotrypsin are commonly employed in vitro digestion models to simulate gastrointestinal degradation of ACE-inhibitory peptides [97,98,99]. Peslerbes et al. simulated gastrointestinal digestion (oral, stomach, and intestine phases) of three plant enzyme hydrolysates and observed that the IC_50_ values of papain- and bromelain-treated hydrolysates were reduced, whereas those of ficin-treated hydrolysates were increased [100]. Research demonstrates that proline residues confer enzymatic stability to bioactive peptides, so active peptides containing proline and hydroxyproline typically exhibit resistance to gastrointestinal proteolysis [101,102]. Ohsawa et al. systematically examined the enzymatic liberation and gastrointestinal stability of the milk-derived tripeptides Val-Pro-Pro (VPP) and Ile-Pro-Pro (IPP) through in vitro digestion models. The study demonstrated that during intestinal digestion, C-terminal proline residues confer enzymatic resistance by impeding carboxypeptidases’ recognition and cleavage of adjacent peptide bonds. As a result, VPP and IPP exhibit high resistance to digestive enzymes [103]. Hui Chen et al. identified a novel oyster-derived peptide (AEYLCEAC) through enzymatic hydrolysis screening. Molecular docking and in vivo experiments demonstrated its competitive ACE inhibition, via active-site hydrogen bonds and significant antihypertensive effects in hypertensive rats models [29].

### 7.2. In Vivo Studies

Bioavailability denotes the proportion of ingested protein available at the vascular level, determined by three main steps: gastrointestinal solubilization and proteolysis, absorption by intestinal epithelial cells, and tissue distribution [99,104]. Consequently, protein bioaccessibility serves as a key parameter determining bioavailability. Given that in vivo experiments account for inter-individual variability, physiological conditions, dosage, and food matrix interactions, bioavailability assessments ultimately require in vivo trials [99]. In vivo evaluation of ACE-inhibitory peptides typically employs spontaneously hypertensive rats (SHRs) through intravenous or intraperitoneal administration or oral gavage to measure systolic blood pressure (SBP) [4,22]. Li et al. administered KYPHVF to SHRs and collected serum, lung, kidney, and liver tissue samples at predetermined time points (0, 1.5, 3.0, and 4.5 h post-administration). The results showed significant blood pressure reduction in SHRs, suggesting that KYPHVF could mediate antihypertensive effects via reducing ACE levels in both serum and renal tissues [105]. Another investigation evaluated the gastrointestinal stability and ACE-inhibitory activity of casein-derived peptides RYLGY, AYFYPEL, and YQKFPQY. RYLGY and AYFYPEL maintained potent ACE-inhibitory activity, with IC_50_ values under 10 μg/mL [106]. Zhou et al. administered four plant- and animal-derived bioactive peptide solutions (corn, wheat, egg white, and soybean) to SHRs and quantified in vivo ACE and Ang-II levels through immunohistochemistry and ELISA. The results showed that both wheat and soybean peptides significantly lowered SBP in SHRs and reduced plasma Ang-II levels [107].

## 8. Conclusions and Future Prospects

Hypertension remains a primary risk factor for cardiovascular and cerebrovascular diseases, with strong associations with diabetes and kidney diseases. Unlike synthetic antihypertensive medications that commonly induce undesirable side effects, food-derived bioactive peptides offer comparable blood pressure-lowering effects with enhanced safety profiles and negligible adverse reactions. Current research on milk-derived peptides has reached relative maturity. Several commercial dairy products containing ACE-inhibitory peptides have been successfully marketed, including Calpis^®^ and Evolus^®^ fermented milk products, as well as specialized formulations such as C12 Peptide^®^ and Lowpept^®^ [108]. Research on plant-derived and marine biological-derived peptides has gained significant attention, owing to their abundant sources and distinctive amino acid profiles [4]. Despite extensive in vitro and computational research on ACE-inhibitory peptides (molecular docking, kinetic simulations), current research still faces three major limitations: a critical lack of human clinical trial validation, complex food matrix interactions, and scalable production [58,100,108]. In summary, future research should prioritize the following: preclinical animal models, development of comprehensive dose–response databases, machine learning-assisted peptide bioactivity prediction, and translational applications in functional food development.

## Figures and Tables

**Table 1:** Comparative analysis with synthetic drug IC50 values.

Synthetic Drug	IC_50_	Reference
Captopril	1.3 × 10^−6^ mg/mL	[18]
Captopril	5 × 10^−4^ μM	[19]
Trandolapril (aortic ACE)	2.5 × 10^−3^ μM	[20]
Trandolapril (renal ACE)	0.015 μM	[20]
Enalapril (aortic ACE)	0.24 μM	[20]
Enalapril (renal ACE)	0.034 μM	[20]

**Table 2:** Production, purification, and characterization of food-derived angiotensin-I-converting enzyme (ACE)-inhibitory peptides.

Substrate		Production, Fractionation, Purification	Condition and Resin/Material	IC_50_	Sequencing and Molecular Mass Determination	Reference
**Plant Sources**	Peony seed	1—Enzymatic hydrolysis 2—Gel filtration chromatography (GFC) and reversed-phase high-performance liquid chromatography (RP-HPLC)	1—Neutral protease 2—Sephadex G-25 column, C18 column, and analytic RP-HPLC column	HWS: 1.38 μM LAGGF: 2.65 μM VLSGF: 0.536 μM LAGYV: 2.80 μM	UPLC-QTOF-MS/MS	[12]
	Ulva prolifera	1—Enzymatic hydrolysis 2—GFC 3—UF	1—Neutral protease (DH: 33.59%) 2—Sephadex-G100 fltration column (4000–15,000 Da)	KAF: 0.63 μM	High-performance liquid chromatography–quadrupole time-of-flight mass spectrometry (HPLC-Q-TOF-MS)	[13]
	Wuyi rock tea residue	1—Alkali solubilization 2—Enzymatic hydrolysis 3—Column chromatography and RP-HPLC	1—0.24 mol/L NaOH (1:34 ratio) 2—Neutral protease 3—Sephadex G-15 column and Phenomenex Gemini C18 column	FPFPRPP: 0.276 μM PPPRGP: 0.801 μM PFPRPPH: 0.369 μM LGHPW: 1.50 μM LKFPDF: 0.517 μML	Ultra-performance liquid chromatography–quadrupole time-of-flight mass spectrometry (UPLC-QTOF-MS/MS)	[21]
	Rice	1—Alkali solubilization 2—Enzymatic hydrolysis 3—RP-HPLC	1—0.1% mol/L NaOH (1:6 ratio) 2—Alcalase and trypsin 3—Cat Ex resin column	-	Multi-angle laser light scattering combined with gel permeation chromatography (MALLS/GPC)	[22]
	Broccoli (brassica oleracea)	1—Water extraction 2—Enzymatic hydrolysis 3—Ethanol extraction 4—GFC, semi-preparative RP-HPLC, and RP-HPLC	1—Hot water (80 °C) 2—Pepsin 3—10% (*v*/*v*) ethanol 4—Sephadex G-15 column, YMC-Pack ODS-AQ column, and C18 column	IAYKPAG: 2.1 μM MRWRD: 0.6 μM MRW: 0.38 μM LRIVA: 4.2 μM	UPLC-QTOF-MS/MS	[23]
	Agaricus bisporus scraps	1—Enzymatic hydrolysis 2—Macroporous resin	1—Alcalase and compound protease 2—DA201-C, XAD1600, XAD7HP, and AB-8	1.50 μM	LC-MS/MS	[24]
	Shiitake mushroom (*Lentinula edodes*)	1—Enzymatic hydrolysis 2—RP-HPLC	1—Alcalase (DH: 28.88%) 2—Luna C18 column	37.14 μM	LC-Q-TOF–MS/MS	[25]
	Microalgal *Chlorella*	1—Enzymatic hydrolysis 2—UF	1—Alcalase 2—3.0 kDa cutoff	2.47 μM–110.2 μM	Q-TOF-LC-MS/MS	[26]
	Walnut	1—Enzymatic hydrolysis 2—HPLC	1—Alcalase (0.5%, *w*/*w*) and papain (0.5%, *w*/*w*) 2—X-Peonyx^®^C18 analytical column	LPVGP: 9.05 µM FPLQPHQP: 5.03 µM	LC-MS/MS	[27]
	Olive pomace	1—Water extraction 2—HPLC 3—GFC	-	2.64–4.59 μM	PAGE, MS	[28]
**Animal Source**	Monkfish (*Lophius litulon*) swim bladders	1—Enzymatic hydrolysis 2—Column chromatography and RP-HPLC	1—Alcalase and neutral protease 2—Sephadex G-25 column	SEGPK: 1.07 μM FDGPY: 1.37 μM SPGPW: 1.16 μM	SDS-PAGE, ESI-Q-TOF-MS	[10]
	Oyster (Crassostrea gigas)	1—Enzymatic hydrolysis 2—HPLC and RP-HPLC	1—Ex vivo digestion 2—ZORBAX Eclipse SB-C18 colum	4287 μM	ESI-Q-TOF-MS	[29]
	*Yamadazyma* spp. in non-fat milk	1—Fermentation 2—HPLC	1—Three yeast strains (BO10, B514-1, and BO13-2) separately and their double and triple combinations 2—C18 column	BO10 and BO13-2: 0.92 mg/Ml BO10 and B514-1: 2.10 mg/mL B514-1 and BO13-2: 2.46 mg/mL	-	[18]
	Skipjack tuna (*Katsuwonus pelamis*) roe	1—Enzymatic hydrolysis 2—UF 3—Column chromatography and RP-HPLC	1—2% (*w*/*w*) flavourzyme 2—1.0, 3.5, and 5.0 kDa cutoffs 3—DEAE-52 cellulose column, Sephadex G-25 column, and Zorbax 300SB-C18 column	WGESF: 1.37 μM IKSW: 1.35 μM YSHM: 0.805 μM WSPGF: 1.01 μM	Protein sequencer, electrospray ionization–quadrupole time-of-flight mass spectrometry (ESI-Q-TOF-MS)	[30]
	*Trichiurus lepturus*	1—Enzymatic hydrolysis 2—UF 3—GFC	1—Alkaline protease 2—3.0 and 10.0 kDa cutoffs 3—Sephadex G-25 column	FAGDDAPRR: 262.98 μM QGPIGPR: 81.09 μM GPTGPAGP: 168.11 μM	LC-MS/MS	[31]
	Crucian carp	1—Enzymatic hydrolysis 2—RP-HPLC	1—Pepsin (4%, *w*/*w*) and trypsin(4%, *w*/*w*) 2—Sephadex G-25 column	GA-Hyp-GAR: 4.00 μM	UHPLC-LTQ-Orbitrap	[32]
	Porcine liver and placenta	1—Enzymatic hydrolysis 2—RP-HPLC	1—Cysteine protease papain 2—Ascentis Express Peptide ES-C18	FWG: 470 μM MFLG: 70 μM SDPPLVFVG: 1160 μM FFNDA: 830 μM	HPLC MS/MS	[19]
	Eel (*Anguilla japonica*) bone collagen	1—Enzymatic hydrolysis 2—UF	1—Alcalase, trypsin, protamex, papain, and pepsin 2—1.0 and 3.0 kDa cutoffs	535.84 μM–3663.82 μM	Nano-HPLC-MS/MS	[33]
	Fermented rubing cheese	1—Aqueous extraction 2—UF	1—Hot water (40 °C) 2—10 kDa cutoff	VAPFPE: 493 μM EKVNELSKD: 98 μM LHLPLPLLQ: 480 μM LQDKIHP: 396 μM	LC-MS/MS	[34]
	Rushan cheese whey	1—Enzymatic hydrolysis 2—UF 3—RP-HPLC	1—Rennet enzyme 2—3.0 and 10.0 kDa cutoffs 3—Thermo Hypersil Gold HPLC	FFVAPFPEVFGK: 52.00 μM VRYL: 24.10 μM YLGY: 41.86 μM TTMP: 51.00 μM RYL: 106.64 μM VYPFPGPIPN: 325.00 μM	MS	[35]

## Data Availability

Data are contained within the article.
